# Comparison of echocardiographic parameters of amputee football players with active football players and sedentary individuals

**DOI:** 10.1186/s13102-023-00651-1

**Published:** 2023-03-24

**Authors:** Ahmet Kurtoğlu, Ertuğrul Kurtoğlu, Nurettin Konar, Bekir Çar, Özgür Eken, Pablo Prieto-González, Hadi Nobari

**Affiliations:** 1 Department of Coaching, Faculty of Sport Science, Bandirma Onyedi Eylul University, Balikesir, 10200 Turkey; 2 Department of Cardiology, Faculty of Medicine, Malatya Turgut Ozal University, Malatya, 44000 Turkey; 3 Department of Physical Education and Sport Teaching, Faculty of Sport Science, Faculty of Sport Science, Bandirma Onyedi Eylul University, Balıkesir, 10200 Turkey; 4 Department of Physical Education and Sport Teaching, Inonu University, Malatya, 44000 Turkey; 5 Health and Physical Education Department, Prince Sultan University, Riyadh, 11586 Saudi Arabia; 6 Department of Exercise Physiology, Faculty of Educational Sciences and Psychology, University of Mohaghegh Ardabili, Ardabil, 56199-11367 Iran; 7 Faculty of Sport Sciences, University of Extremadura, Cáceres, 10003 Spain

**Keywords:** Amputation, Echocardiography, Cardiac dysfunction

## Abstract

**Background:**

The purpose of this study is to compare the echocardiographic (ECHO) parameters of amputee football players (AF) with those of athletes without a disability (football players) (FP), and sedentary individuals (SI).

**Methods:**

A total of 37 male participants (nAF = 12, nFP = 12, nSI = 13) were included in the study. All participants underwent a transthoracic echocardiographic examination. Aortic diameter in systole (ADs), aortic diameter in diastole (ADd), isovolumic contraction time (IVCT), isovolumic relaxation time (IVRT), left ventricular ejection fraction (LVEF), early (E) and late (A) wave velocities, myocardial systolic (S), early diastolic (E’), and late diastolic (A’) myocardial rates, interventricular septal thickness (IVS), left ventricular end-diastolic diameter (LVDd) and left ventricular end-systole diameter (LVDs), left ventricular posterior wall thickness (LVPWd), left atrial diameter (LAD), and ascending aortic diameter (AAD) were measured.

**Results:**

LVDd, E’ were lower in AF than in FP. In contrast, LVDs, LVPWd, and A wave were higher in AF than in FP. When AF and SI groups were compared, ADs, LVPWd, A wave, IVRT, and S wave were higher in AF than in SI. ANOVA test showed a statistically significant difference between groups in LVPWd, A-wave, and E’ wave. TTE data indicate that some parameters in AF differ from those observed in healthy individuals. The smaller LVEED diameter and higher PWT were found in AF.

**Conclusions:**

Although within normal limits, some ECHO parameters in the AF group differed from those without disability. Future studies should further investigate these differences using different and detailed measurement methods.

## Introduction

Athlete’s heart syndrome is electrophysiological, structural, and functional myocardial adaptations associated with sustained training stimuli without pathological significance [1]. Depending on this syndrome, 10–15% enlargement in the left and right ventricles of the athletes and an increase in the left ventricular wall thickness by 10–20% can be observed. Functionally, an increase in stroke volume and improvement in cardiac filling in diastole, and improvement in capillary conductance and skeletal muscle oxidative capacity may be observed in athletes [2]. Echocardiography is an important method for determining heart function in patients and athletes is the method of using ultrasound (ultrasonic) waves to diagnose cardiovascular disorders [3]. Evaluation of heart chambers size and volume, left ventricular diastolic and stroke volume, cardiac output and left ventricular ejection fraction (EF), and the heart’s pumping function can be easily measured by echocardiography. Thus, it plays an important role in heart disease diagnosis and monitoring. It also provides information about heart width [4]. When studying athletes’ cardiac morphology, it was found that there was a moderate association between eccentric and concentric left ventricular hypertrophy compared to mixed sports activities and training protocols [5].

Amputation, which occurs for different reasons, also causes changes in the cardiovascular system. Resnik et al. determined that 26% of individuals who had lower extremity amputation due to various reasons died from cardiovascular diseases [6]. In the study conducted by Flank et al, it was reported that the body mass indexes of individuals who are wheelchair dependent after spinal cord injury increase and this situation triggers diseases such as hypertension and diabetes [7]. Also, amputation of a limb can cause some problems due to a change in the center of gravity, impaired walking and running abilities, high energy expenditure, increased heart rate, and decreased oxygen consumption [8]. For these reasons, it is thought that cardiovascular system markers should be checked at frequent intervals in sports branches that require endurance such as amputee football.

Recent studies have found that high-level endurance training damages cardiac biomarkers and decreases left ventricular function [9–13]. In this context, high-dose training applications in AF players could cause problems for the cardiovascular system due to high heart rate and amputation-related peripheral arterial pressure [14]. In a study by Bernardi et al in which they compared the cardiovascular risk factors of paralympic athletes with spinal cord injury, upper extremity disorder, and lower extremity amputation, it was reported that the cardiometabolic risk factor was higher in individuals with lower extremity amputation compared to other groups [15]. There are many studies in the literature comparing amputee athletes competing in paralympic sports with other disabled athletes. However, no study was found in which the ECHO parameters of AFs were compared with non-disabled athletes and healthy individuals. Therefore, we aimed to investigate cardiac dimensions and cardiac systolic and diastolic functions of AF players using transthoracic echocardiography (TTE) compared to their healthy and sedentary counterparts. The hypothesis of our research was determined as ‘the echocardiographic parameters of amputee football players are different from healthy athletes without a disability and sedentary individuals.

## Materials and methods

### Study design

The experimental method —one of the quantitative data collection techniques — was used in the present study [16]. Malatya Metropolitan Municipality Amputee Football Team players, Malatya Amateur League football players, and sedentary individuals participated in our study. Participants were selected on a voluntary and random basis. Before the study, TTE examinations were performed by a cardiology specialist after taking anthropometric measurements such as age, height, weight, and BMI of the participants. Participants were asked not to do any exercise that could affect the results 48 hours before TTE. All measurements were made before the season and after at least 8 hours of complete passive rest. The necessary permissions for this study were obtained from the management of the Turkish Physical Handicapped Sports Federation and Malatya Amateur Football Clubs Federation. Exclusion criteria from our study are a) with hypertension, b) diabetes, c) coronary artery disease, e) cardiac arrhythmias, f) thyroid disease, h) moderate to severe valvular heart disease, ı) active infections, and j) smoking. Inclusion criteria in our research are a) playing in the amputee football league for at least 1 year, playing active football in any football league for at least 1 year, c) and be a healthy sedentary with less than 2 hours of exercise per week. Participants who a) used performance-enhancing drugs after our research began, and b) not following the principal investigator’s instructions were excluded from the study. The study was conducted under the provisions of the Declaration of Helsinki. The ethical approvals required for this study were obtained from the Bandirma Onyedi Eylul University, Ethics Committee of the Institute of Health Sciences (Approval Nummer: 2022-55).

### Participants

The study population consisted of amputee football players (AF), non-disabled athletes (FP), and sedentary (SI) men. AF subjects were selected from a group of elite amputee football players, and FP players were selected from amateur league football teams. Moreover, the SI participants included were subjects who did not have chronic diseases, and their weekly exercise duration was < 2 hours. Web-Based Sample Size & Power Analysis Software (WSSPAS) was used to determine the number of participants to be included in the study [17]. Analyzing the type I error (α) as 0.05 and power (1-β) as 0.80, it was found that at least 10 participants were required for each AF, FP, and SI group. The baseline characteristics of the studied population are shown in Table 1.

**Table 1:** Identifiable information about groups

Parameters	AF	FP	SI
**Age (y)**	24.58 ± 5.07	23.08 ± 4.40	23.65 ± 5.05
**Weight (kg)**	70.42 ± 14.42	71.42 ± 9.19	74.77 ± 16.61
**Height (cm)**	175.75 ± 10.14	178.33 ± 6.58	181.23 ± 6.54
**BMI(kg/m** ^**2**^ **)**	22.79 ± 4.01	22.40 ± 2.07	22.69 ± 4.33
** h (beats/min)**	81.66 ± 13.36	76.45 ± 12.46	73.08 ± 9.78
**SBP (mmHG)**	122.91 ± 3.84	124.41 ± 3.20	124.07 ± 3.75
**DBP (mmHG)**	81.16 ± 2.12	80.66 ± 3.57	80.38 ± 2.53

BMI: Body Mass Index, (Data was given as means with standard deviation), y: year, HR: Hearth Rate, SBP: Systolic Blood Pressure, DBP: Diastolic Blood Pressure

### Data collection

TTE evaluation of the study participants engaged in sports was conducted without season training. All measurements of the participants were taken out of season. The clinical findings of all participants were analyzed by the cardiologist. All participants exhibited normal ECG findings. At the same time, the systolic and diastolic blood pressures of the participants were within normal limits. After obtaining the necessary permissions for the research, all the study participants underwent TTE evaluations at the cardiology department. TTE was performed with a Vivid S5 echocardiography machine with an S3 transducer by two cardiologists who were blind to the clinical characteristic of each subject. TTE was performed with a Vivid S5 echocardiography machine (GE, Boston, USA). During TTE examinations, subjects removed their clothing at the waist in dim light and lay in the left lateral decubitus position. The following 2-dimensional and M-mode echocardiographic parameters were measured: aortic diameters both in systole (ADs) and diastole (ADd), left ventricular end-diastolic diameter (LVEDD, mm), left ventricular end-systolic diameter (LVESD, mm), left atrial diameter (LAD, mm), interventricular septal thickness (IVST, mm), and posterior wall thickness (PWT, mm). Transmitral pulsed-wave Doppler velocities were measured from the apical 4-chamber view with a Doppler sample placed at the level of the mitral valve leaflet. Early (E-wave, m/s) and late (A-wave, m/s) flow velocities, ejection time (ET, ms), isovolumic relaxation time (IVRT, ms), isovolumic contraction time (IVCT, ms) were measured. Tissue Doppler imaging of annulus motion was measured from lateral mitral annulus and peak early systolic (Sm, m/s), peak early diastolic (Em, m/s), and peak late diastolic (Am, m/s) velocities. Left ventricular ejection fraction (LVEF) was calculated using Simpson’s method. Each LV mass was calculated and indexed to the body surface area. Left ventricular mass was calculated using a simple and anatomically validated formula: LVM = 0.8 × 1.04 [(IVS + LVEDD + LVPW)^3^ − LVEDD^3^] + 0.6 [18]. Relative Wall Thickness (RWT) is measured as RWT = 2x(PWT/LVEDd) [19].

### Aortic strain and distensibility

Aortic vessel strain and distensibility values were determined using the following formulas [20]. Aortic strain (AS)=(aortic systolic diameter-aortic diastolic diameter)/(aortic diastolic diameter) × 100. Aortic distensiblity (AD) = 2xAS/(aortic systolic diameter-aortic diastolic diameter).

### Intraobserver and interobserver variability

The absolute mean difference ± SD between measurements, within one observer and between two observers, of AA 2D STI LS were 1.3 ± 1.2% and 1.6 ± 1.3%. The intraobserver and interobserver ICCs were 0.889 and 0.852, respectively.

### Statistical analysis

The statistical operations performed in the study were carried out with the SPSS package program 25. The normality analysis of the data was tested with the Shapiro-Wilk test since the number of participants < was 50. The data distribution and homogeneity of variance were checked using Shapiro-Wilk and Levene tests respectively. To analysis of data the Shapiro-Wilk and Levene tests were used to evaluate the normality and homogeneity of variances. The results were reported as mean ± standard deviation, with a 95% of confidence interval (CI). It was found that the data were not normally distributed, and the nonparametric Mann-Whitney U test was used for pairwise comparisons, and the Kruskal-Wallis H test was used for three-group comparisons. The effect sizes were calculated and classified to determine the magnitude of changes among the experimental conditions as proposed by ‘Cohen’s d’. An effect size classified as 0.2 was deemed small, 0.5 as medium, and 0.8 as large [21]. The significance level in the study was set as p < 0.05.

## Results

A total of 37 subjects were analyzed in the current study. AF group included 12 subjects, FP group 12 subjects, and the SI group 13 subjects. The results of TTE variables are presented in Table 2, AF group showed statistically significant higher values of LVESD (p = .037, ES = 0.823), PWT (p = .031, ES = 0.978), and A-wave (p = .019, ES = 0.942) values than those of FP group, while LVEDD (p = .026, ES = 1.024), Em (p = .031, ES = 784), and E/A ratio showed lower values in AF group than the FP group. ADs (p = .038, ES = 0.786), PWT (p = .048, ES = 0.243), A wave (p = .024, ES = 0.941), IVRT (p = .020, ES = 1.044) Sm wave (p = .047, ES = 0.266) were also found statistically to be higher in the AF group than in SI group. The only statistically different parameter between FP and SI groups was Em, which showed higher values in the FP group. There was no difference between the groups in other ECHO parameters (p > .05).

**Table 2:** Mann Whitney U Test Results of Groups

Parameter	Group	x̄ ± ss	EF	*p*	Group	x̄ ± ss	EF	*p*	Group	x̄ ± ss	Z	*p*
ADs (mm)	AF	27.99 ± 3.77	0.607	0.192	AF	27.99 ± 3.77	0.786	0.038*	FP	26.00 ± 2.69	0.272	395
	FP	26.00 ± 2.69			SI	25.15 ± 3.46			SI	25.15 ± 3.46		
LVEDD (mm)	AF	45.50 ± 6.30	1.024	0.026*	AF	45.50 ± 6.30	0.632	0.126	FP	50.91 ± 4.01	0.507	0.209
	FP	50.91 ± 4.01			SI	48.84 ± 4.14			SI	48.84 ± 4.14		
LVESD (mm)	AF	35.09 ± 4.41	0.803	0.037*	AF	35.09 ± 4.41	0.188	0.623	FP	32.08 ± 2.93	0.631	0.139
	FP	32.08 ± 2.93			SI	34.30 ± 3.98			SI	34.30 ± 3.98		
PWT (mm)	AF	10.00 ± 1.41	0.978	0.031*	AF	10.00 ± 1.41	0.243	0.048*	FP	8.75 ± 1.13	0.256	0.527
	FP	8.75 ± 1.13			SI	9.00 ± 0.81			SI	9.00 ± 0.81		
A-Wave (m/s)	AF	0.58 ± 0.09	0.942	0.019*	AF	0.58 ± 0.09	0.941	0.024*	FP	0.48 ± 0.12	0.197	0.479
	FP	0.48 ± 0.12			SI	0.50 ± 0.08			SI	0.50 ± 0.08		
E/A Ratio	AF	1.44 ± 0.28	0.981	0.024*	AF	1.44 ± 0.28	0.555	0.301	FP	1.73 ± 0.31	0.392	0.446
	FP	1.73 ± 0.31			SI	1.60 ± 0.35			SI	1.60 ± 0.35		
IVRT (ms)	AF	62.91 ± 9.86	0.725	0.132	AF	62.91 ± 9.86	1.044	0.020*	FP	54.91 ± 12.08	0.192	0.460
	FP	54.91 ± 12.08			SI	52.61 ± 11.87			SI	52.61 ± 11.87		
Em (m/s)	AF	0.16 ± 0.02	0.784	0.031*	AF	0.16 ± 0.02	0.000	0.527	FP	0.18 ± 0.03	0.796	0.035*
	FP	0.18 ± 0.03			SI	0.16 ± 0.02			SI	0.16 ± 0.02		
Sm (m/s)	AF	0.12 ± 0.05	0.525	0.143	AF	0.12 ± 0.05	0.266	0.047*	FP	0.10 ± 0.02	0.5	0.866
	FP	0.10 ± 0.02			SI	0.11 ± 0.02			SI	0.11 ± 0.02		
AST	AF	0.12 ± 0.05	0.181	0.525	AF	0.12 ± 0.05	0.838	0.157	FP	0.13 ± 0.06	0.727	0.041*
	FP	0.13 ± 0.06			SI	0.09 ± 0.05			SI	0.09 ± 0.05		

ADs: Aortic diameter systole, AST: Aortic strain, Em: Peak early diastolic, IVIVRT: Isovolumic relaxation time, LVEDD: Left ventricular end-diastolic diameter, LVESD: Left ventricular end-systolic diameter, PWT: Posterior wall thickness, Sm: Peak early systolic

As a result of the ANOVA test in Table 3, a statistically significant difference was found between the PWT (AF > SI > FP, p < .05), A-Wave (AF > SI > FP, p = .025), E’ (SI > AF > FP, p < .050) and Em values (FP > SI > AF, p = .043) of the participants.

**Table 3:** Kruskal Wallis H Test Results of Groups

Parameter	AF Group (n = 12)	FP Group (n = 12)	SI Group (n = 13)	ES	p
AA (mm)	23.58 ± 1.50	24.08 ± 2.96	23.46 ± 1.80	0.025	0.988
LAD (mm)	32.00 ± 3.07	32.55 ± 3.01	31.46 ± 5.34	0.625	0.731
ADs (mm)	27.99 ± 3.77	26.00 ± 2.69	25.15 ± 3.46	4.559	0.102
ADd (mm)	24.95 ± 3.81	22.91 ± 3.02	24.15 ± 3.67	2.611	0.271
AD	0.081 ± 0.012	0.088 ± 0.011	0.084 ± 0.012	2.611	0.271
LVEDD (mm)	45.50 ± 6.30	50.91 ± 4.01	48.84 ± 4.14	5.879	0.053
LVESD (mm)	35.09 ± 4.41	32.08 ± 2.93	34.30 ± 3.98	4.512	0.105
IVST (mm)	9.33 ± 1.37	8.58 ± 1.24	8.92 ± 1.93	1.569	0.456
PWT (mm)	10.00 ± 1.41	8.75 ± 1.13	9.00 ± 0.81	5.981	0.050*
RWT	0.44 ± 0.08	0.34 ± 0.04	0.37 ± 0.04	8.776	0.001*
E-Wave (m/s)	0.83 ± 0.14	0.82 ± 0.14	0.79 ± 0.14	0.958	0.619
A-Wave(m/s)	0.58 ± 0.09	0.48 ± 0.12	0.50 ± 0.08	7.366	0.025*
E/A Ratio	1.44 ± 0.28	1.73 ± 0.31	1.60 ± 0.35	4.407	0.110
ET (ms)	269.18 ± 34.73	270.75 ± 22.15	269.25 ± 18.66	1.526	0.466
IVCT (ms)	69.08 ± 18.87	57.75 ± 12.07	62.41 ± 11.71	3.990	0.136
IVRT (ms)	62.91 ± 9.86	54.91 ± 12.08	52.61 ± 11.87	5.547	0.062
Em (m/s)	0.16 ± 0.02	0.18 ± 0.03	0.16 ± 0.02	6.298	0.043*
Am (m/s)	0.09 ± 0.02	0.08 ± 0.01	0.09 ± 0.02	2.351	0.309
Sm (m/s)	0.12 ± 0.05	0.10 ± 0.02	0.11 ± 0.02	4.135	0.127
Em/Am Ratio	1.82 ± 0.58	2.35 ± 0.73	1.80 ± 0.48	4.600	0.100
E/Em Ratio	5.26 ± 0.94	4.51 ± 1.10	4.85 ± 0.84	3.572	0.168
LVEF (%)	54.50 ± 5.69	56.67 ± 7.24	59.70 ± 6.63	1.804	0.320
AS	0.12 ± 0.05	0.13 ± 0.06	0.12 ± 0.05	4.484	0.106
LVM (g)	151.68 ± 46.37	156.93 ± 38.69	152.63 ± 37.37	0.057	0.945
LVMI (g/m^2^)	85.13 ± 24.20	83.25 ± 17.22	79.01 ± 16.66	0.323	0.726

AA: Ascending aortic diameter, AD: Aortic distensibility, ADd: Aortic diameter diastole, ADs: Aortic diameter systole, Am: Peak late diastolic, AS: Aortic strain, Em: Peak early diastolic, ET: Ejection time, IVCT: Isovolumic contraction time, IVST: Interventricular septal thickness, IVRT: Isovolumic relaxation time, LAD: Left atrium diameter, LVEDD: Left ventricular end-diastolic diameter, LVEF: Left ventricular ejection fraction, LVESD: Left ventricular end-systolic diameter, LWM: Left Ventricular Mass, LVMI: Left Ventricular Index, PWT: Posterior wall thickness, RWT: Relative Wall Thickness, Sm: Peak early systolic

**Fig. 1:**
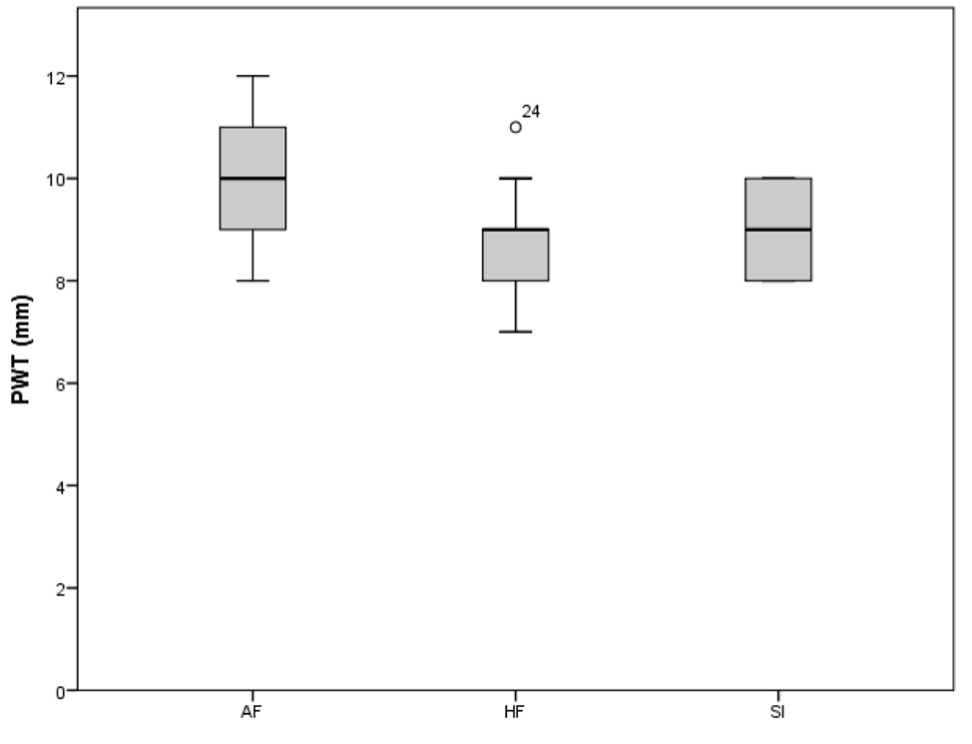
Comparison of PWT levels of groups. A significant difference between AF and other groups (FP, SI) (p < .05)

When Fig. 1 was examined, it can be observed that PWT levels were higher in favor of AF (p < 0.05). Although there was a significant difference between AF and FP at the level of p = 0.031 and between AF and SI at the level of p = 0.048, no difference was found between FP and SI.

**Fig. 2:**
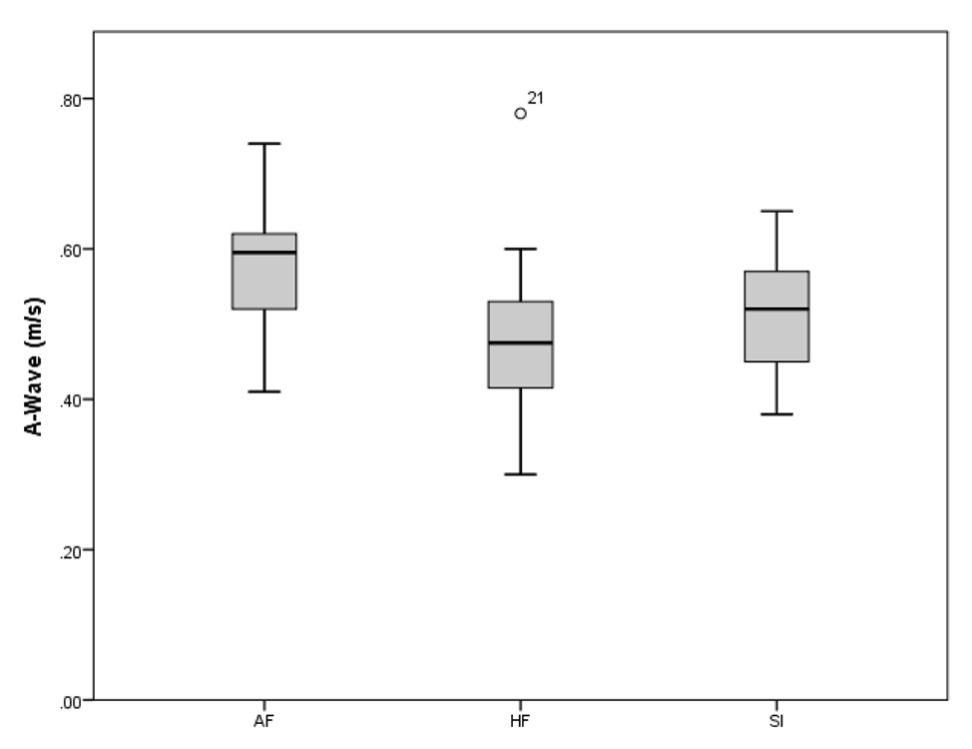
Comparison of A-Wave levels of groups. A significant difference between AF and other groups (FP, SI) (p < .05)

Figure 2 shows that A-Wave levels were higher in favor of AF (p < 0.05). Although there was a significant difference between AF and FP at the level of p = 0.019 and between AF and SI at the level of p = 0.024, no difference was found between FP and SI (p > 0.05).

**Fig. 3:**
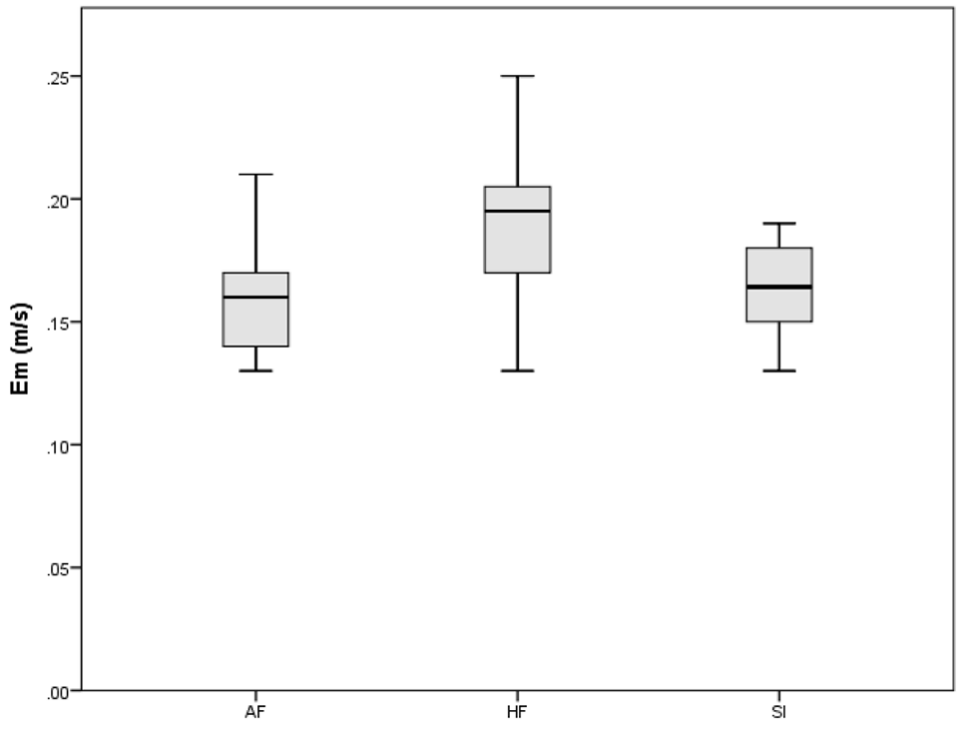
Comparison of Em levels of groups. A significant difference between FP and other groups (AF, SI) (p < .05)

Em levels were low in favor of AF (see Fig. 3). A significant difference between AF and FP was found at the level of p = 0.031 and 0.035 between FP and SI. No significant difference was detected between AF and SI (p > 0.05).

## Discussion

Although the ventricular diameters of all participants were within normal limits, LVEDD was lower in the AF group. This difference might be due to the thicker PWT in the AF group. In addition, it was found in our study that the A wave was higher in the AF group. Tissue Doppler parameters, Em being not different between groups and Am being large in favor of AF indicate that amputation produced some diastolic changes in the heart. This study is the first to compare the echocardiographic parameters of amputees and healthy subjects.

The importance of diastole to cardiac function in terms of cardiac output and exercise capacity has become clearer in recent years. Isolated diastolic dysfunction is common in the elderly, persons with diabetes and vascular disease, and persons with left ventricular hypertrophy [22]. The rate of diseases such as aortic root dilatation, valvular diseases, and hypertension in disabled athletes is not to be underestimated [23]. However, there is evidence that patients with normal diastolic function at rest may have diastolic dysfunction during exercise [24, 25]. In the study by Jensen-Urstad et al., wherein the post-competition cardiac values ECHO of middle and long-distance runners were evaluated, LVEDD increased by 14% in the runners and 12% in the control group [26]. In another study, Massoure et al. found that LVEDD and LVESD levels increased in endurance athletes after peak training compared to the control group [27]. In this context, it was concluded that increased ventricular diameters after exercise is expected [28]. In a study conducted by Park et al. in which middle and long-distance runners and weightlifters compared values at rest and after exercise ECHO, it was found that LVEDD, LVESD, IVST, and PWT at rest were higher in middle and long-distance runners [29]. When comparing the research results in the literature and the findings obtained in our study, it was found that acute and chronic endurance studies caused an increase in ECHO parameters such as PWT and LVEDD.

In the study by Pelliccia et al, the ECHO parameters of paralympic athletes with and without spinal cord injury (SCI) were examined and it was determined that athletes with spinal cord injury had smaller LVEDD. In addition, a smaller LV cavity and LVM were found due to lower physical performance in athletes with SCI. Parasport athletes who are not SCI were found to have a larger cardiac girth during exercise because they used a larger muscle mass [30]. In our study, LVM was lower in favor of AF, although there was no significant difference. The lack of difference in LVM and LVMI in the AF group might be related to exercise-induced cardiac remodeling, as described in previous studies [14].

However, in our study, ECHO evaluations of all participants were performed after at least 8 hours of rest. However, it was found that LVEDD was lower in the AF group. At the same time, PWT was higher in the AF group than in the other groups. The low LVEDD in the AF group might be due to the high PWT. When these results in the AF group are compared with those in the FP and SI groups, diastolic dysfunction might be present in the AF group.

The Em rate decreases when cardiac relaxation is disturbed (diastolic dysfunction). After early relaxation, the ventricular myocardium is passive, and the late rate peak Am is a function of atrial contraction. In addition to absolute values, the ratio between Em and Am is also a measure of diastolic function [31, 32]. In mild diastolic dysfunction, the peak velocity of early mitral flow E wave decreases proportionally to Em. However, when it decreases at the moment of relaxation to the point where there is an increase in atrial pressure, the E wave increases again, whereas Em remains low due to a lower strain. From this point of view, the E/Em ratio is related to atrial pressure and may indicate increased filling pressure [33]. In the study by Ardahanlı et al. in which they compared the ECHO results of patients with mild and moderate covid-19 disease and healthy subjects, although no significant difference was found in the ECHO parameters between the two groups, E and A waves were higher in patients diagnosed with covid-19 [34]. Anderson et al. examined diastolic cardiac functions after 16 weeks of soccer and jogging training in healthy sedentary women. They found an increase in the E wave and a decrease in the A wave in both groups [35]. Considered in this context, regular exercise has a positive effect on diastolic parameters [36]. However, our study found that the A wave was significantly higher in the AF group than in the other groups. At the same time, Am, one of the tissue Doppler parameters, was significantly higher in the AF group. This may indicate diastolic dysfunction in the AF group.

Regular exercise has long been known to affect cardiac structure [37]. It is known that an increase in heart rate also causes ECHO parameters such as LVEDD, LVESD, and EF to beat [38, 39]. Endurance and aerobic resistance training have been found to significantly increase LVED, LVESD, ADd, ADs, and EF and decrease ADs [22, 23]. When the TTE scores of well-trained football players were compared with the control group, their E-wave, A-wave, ET, and IVCT were high, and their IVRT was low [40]. Evidence shows that AD varies with age, athletic age, and training status. Ryffel’s study found that middle-aged distance runners have a higher ADI than lower age groups. However, AS decreases with increasing years of training [41]. Cesareo et al. found that AS is associated with ascending aortic dilatation in hypertensive individuals [42]. Churchill et al. found that aortic dilation is common in various endurance athletes [43]. Durmaz et al. found that AS is positively and significantly associated with atherosclerosis [20]. By examining the literature, it was found that long-distance running, endurance training, and resistance exercise affects TTE parameters. The lack of difference in these parameters in our study might be the type of exercises performed.

This research was carried out after the amputee and amateur football season and the chronic effect of exercise was tried to be determined. The same study can be done during the season, and exercise’s acute effect on cardiac markers can be examined. This is one of the limitations of our research. Another limitation of our study is that participants’ left atrial functions (LAF) were not determined. In future studies, more meaningful results can be obtained by determining LAF in individuals with AF. Besides, the heart structures of individuals with amputation can be analyzed by different methods. Studies can be conducted on the effects of the cardiovascular system in amputee individuals in preparation for exercise, during exercise, and in recovery. At the same time, the effects of amputation rate (transtibial, transfemoral, knee articulation, hip articulation, etc.) on the cardiovascular system can also be examined.

## Conclusions

It has been reported in previous studies that the level of cardiorespiratory fitness decreases with amputation and this is normalized with appropriate physical activities [44]. Although ECHO parameters in the AF group were within normal limits, TTE data show that some parameters were different from healthy individuals. The lower LVEDD diameter, higher PWT, and higher detection of Am in the A wave in the AF group indicated the need for a detailed study of cardiac parameters in amputees. Therefore, the results of this study can be a reference for future research to determine the causes of these cardiac differences in amputees. Similarly, the findings obtained may be helpful for coaches interested in amputee soccer to determine the content of their training programs accordingly.

## Data Availability

The data presented in this study are available on request from the corresponding author.

## Abbreviations

ECHO: Echocardiographic
AF: Amputee football players
FP: Healthy football players
SI: Sedentary individuals
ADs: Aortic diameter in systole
ADd: Aortic diameter in diastole
IVCT: Isovolumic contraction time
IVRT: Isovolumic relaxation time
LVEF: Left ventricular ejection fraction
E: Early
A: Late
S: Myocardial systolic
E': Early diastolic
A': Late diastolic
IVS: Interventricular septal thickness
LVDd: Left ventricular end-diastolic diameter
LVDs: Left ventricular end-systole diameter
LVPWd: Left ventricular posterior wall thickness
LAD: Left atrial diameter
AAD: Ascending aortic diameter
